# Supplementary material for: The Impact of Alcohol Consumption and Oral Microbiota on Upper Aerodigestive Tract Carcinomas: A Pilot Study
Source: Antioxidants (Basel). 2023 Jun 7;12(6):1233. doi: 10.3390/antiox12061233 (PMC10295395; doi:10.3390/antiox12061233)
Supplement: Supplementary file 1 [file antioxidants-12-01233-s001.zip › antioxidants-2425143-supplementary.pdf]

Supplementary Material

Primer for PCR, cycle-sequencing and minisequencing

The free online software Primer3web version 4.1.0 (https://bioinfo.ut.ee/primer3-0.4.0/) was used to design oligos and probes; as input, the DNA sequence containing the SNPs of interest was used. DNA sequences of forward strand of ADH7, ALDH2, ADH1B and ADH1C genes were obtained from Ensembl genome browser (https://www.ensembl.org/index.html). The same primer pair for PCR (polymerase chain reaction) was used for amplification and forward and reverse sequencing (Table S1). The list of the analyzed SNPs, and the related probes used for genotyping by minisequencing are shown in Table S2.

Table S1. Forward and reverse primer sequences used to amplify regions of interest and length of the generated amplicons.

Name	Sequence	Name	Sequence	Amplicon Lenght
**F1**	5'-TGAAAGCCATGCACACTAGC-3'	**R1**	5'-GAGAAGACCAAAACTAGAGGAGC-3'	581bp
**F2**	5'-GGGCAAGTAAAAGGGTCCCC-3'	**R2**	5'-ACCTCAGATGCTCCTTGGAC-3'	476bp
**F3**	5'-TCGTCTCTCATTGCCTTGGT-3'	**R3**	5'-GGGAAGGTAGAGAAGGGCTT-3'	417bp
**F4**	5'-GCTTAGCTGATTGAATGGAAGAC-3'	**R4**	5'-TGCTGTGGACATCAACAAGG-3'	351bp
**F5**	5'-CTCACTGTATGCCTGCTCAC-3'	**R5**	5'-GCCAGACTATCTATGTGAAGGC-3'	273 bp
**F6**	5'-AGCATTGCATTCTCTACATTGTG-3	**R6**	5'-GAGAGGGATCTTGTCTGATGGT-3'	683bp
**F7**	5'-TACATAGCAGGCAAAGGACC-3'	**R7**	5'-AGTGCTGTGATGACATCTGC-3'	510bp
**F8**	5'-ACCATGGTTTCAAGATGCCC-3'	**R8**	5'-TTCCACTTGCGTCGTCTTTG-3'	215bp
**F9**	5'-GCTACAAGATGTCGGGGAGT-3'	**R9**	5'-CCCCAACAGACCCCAATCC-3'	165bp
**F10**	5'-ATCGTCAGAGTGAACAGGCA-3'	**R10**	5'-CACTTTTGGATGGGGTTCTTCA-3'	141bp

Table S2. Mini-sequencing primers and their sizes in bp. Sequence: in black, the annealing sequence ending at the base preceding the SNP of interest; in red, the polynucleotides non hybridizing tail that was added as an electrophoretic mobility modifier. Primer length: indicates the number of bases composing the probe (in brackets the length of the non-hybridizing tail). Strand target: plus, or minus refers to the strand to which the probe is annealing.

	Name	Gene	Polymorphism	Sequence	Strand target	Primer Lenght	Pmol in Mix assembly
Mix3	MS1	ADH1B	rs1229984	TGGTGGCTGTAGGAATCTGTC	Plus	21	5
	MS2	ADH1B	rs2066702	GTCTCTTCTTTCCTATTGCAGTATC	Plus	25	5
	MS3	ADH1C	rs1693482	AGAGAAAGGATCATACCATGGTGTCAAGC	Minus	29 (7)	5
	MS4	ADH7	rs894363	AAGAAGCTTTTGTCCCCAAATAACTCTAC TTTC	Minus	33 (6)	5
	MS5	ADH7	rs1154470	AAAGGAAAAAGTCTAAGTATCAGCCTCAC ATCATTAG	Minus	37 (11)	1.25
Mix4	MS6	ALDH2	rs671	GGCTGCAGGCATACACT	Minus	17	0.6
	MS7	ADH7	rs1154468	AAAAGAAAAGGAAAGAAAGAAACATGCC ACAACATCCTTAA	Plus	41 (19)	1.25

MS8	ADH7	rs1573496	AGGAGAAGGGAAAAAAGAAATATACCTG GTTTCACTGTAGTCACT	Minus	45 (19)	1.25
MS9	ADH7	rs971074	AAGAAAAAGGAAGAAAAGAGGGAGATTT GTTGAGGTCAATCCCAATGAT	Minus	49 (25)	1.25
MS10	ADH7	rs1154461	AAAGAGAAGAAAAAGAAAAAAAAAAAA GACAGAATGGGAGGAGATTTTTGTGAT	Minus	54 (25)	1.25

Multiplex-PCR

Amplicons containing the regions of interest were divided in two multiplex PCRs, each amplifying five amplicons differing in molecular weight. Amplicons were distinguished by their size using a 50 bp molecular weight standard (Thermo Fisher Scientific, Waltham, Massachusetts, USA) running 3.3 µL of each sample in a 1.5% agarose gel electrophoresis (BioRad, Hercules, California, USA) (Figures S1, S2). Primer MIX1 amplicons contained the following SNPs (in brackets amplicon length): rs894363 (581bp), rs2066702 (476bp), rs1229984 (417bp), rs1693482 (351bp) and rs1154470 (273bp). MIX2 amplicons contained: rs1573496 (683bp), rs1154468 (510bp), rs971074 (215bp), rs671 (165bp) and rs1154461 (141bp) (Table S3).

PCRs were performed in a PTC100 Thermal Cycler (Bio-Rad, Hercules, California, USA) in a reaction volume of 15 µL. The reaction mix was assembled as follow: 3.35 µL of tetradistilled water, 2.1µL of deoxynucleotides (dNTP) 1.25 mM (Thermo Fisher Scientific, Waltham, Massachusetts, USA), 3 µL of Buffer Mix Reaction 5x (Promega, Madison, Wisconsin, USA), 0.9 µL of magnesium chloride 25 mM (Promega, Madison, Wisconsin, USA), 0.6 µL of the primer mix (Sp - Table 3) and 0.05 µL of Gotaq DNA polymerase 5 U/mL (Promega, Madison, Wisconsin, USA) and 5 µL of genomic DNA (10 ng).

Table S3. Primers used for polymorphism analysis: primers pairs composing the two mixes, assembled from 100 pmol/µL solutions and related reaction conditions. Ta= annealing temperature (in common between primer pairs composing the mix).

	Name	Gene	Polymorphism	Ta	pmol in PCR
	F1-R1	ADH7	rs894363		3.5
	F2-R2	ADH1B	rs2066702		6
**MIX1**	F3-R3	ADH1B	rs1229984	56°C	3.5
	F4-R4	ADH1C	rs1693482		6
	F5-R5	ADH7	rs1154470		11.5
	F6-R6	ADH7	rs1573496		6
	F7-R7	ADH7	rs1154468		3
**MIX2**	F8-R8	ADH7	rs971074	61°C	3
	F9-R9	ALDH2	rs671		2
	F10-R10	ADH7	rs1154461		6

The mix1 PCR protocol included the following steps: 95° C for 2’; (94° C for 45’’; 56° C for 1’30’’; 72° C for 2’30’’) for 38 cycles; 72° C for 7’; 10° C for ∞.

The mix2 PCR protocol included the following steps: 95° C for 2’; (94° C for 45’’; 61° C for 1’30’’; 72° C for 2’30’’) for 40 cycles; 72° C for 7’; 10° C for ∞.

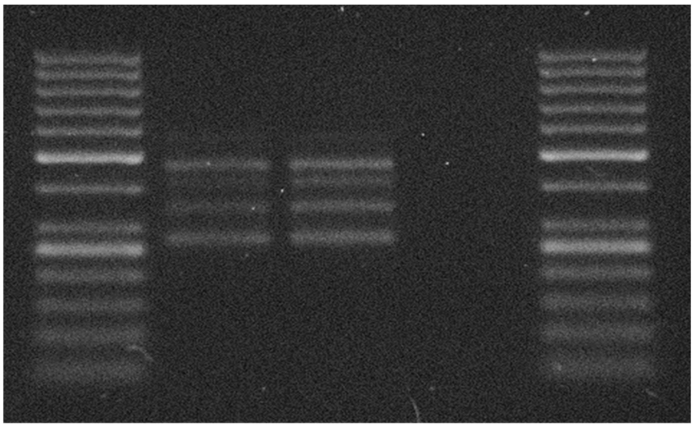

Figure S1. Electrophoresis on 1.5% agarose of mix1. Lanes 1 and 5, Ladder 50 bp Thermo Fisher (Waltham, MA, USA). Lane 2, DNA 1. Lane 3, DNA 2. Lane 4, negative control.

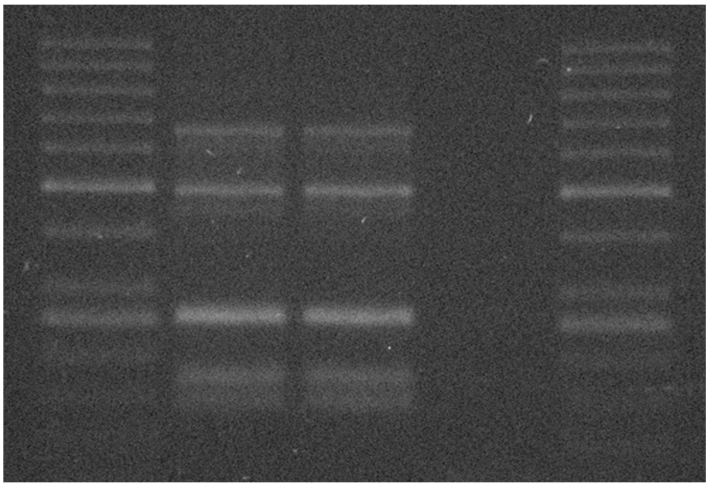

Figure S2. Electrophoresis on 1.5% agarose of mix2. Lanes 1 and 5, Ladder 50 bp Thermo Fisher (Waltham, MA, USA). Lane 2, DNA1. Lane 3, DNA2. Lane 4, negative control.

Purification of PCR

An enzymatic purification was performed adding to each PCR sample 1.2 μL of FastAP (Thermosensitive Alkaline Phosphatase), 0.6 μL of Exonuclease I and 1.5 μL of 10x Reaction Buffer for Exonuclease I buffer (all from Thermo Fisher Fisher, Waltham, MA, USA) in a total volume of 15 μL. Samples were then incubated at 37° C for 60’ (activation of the enzymes) and then at 80° C for 15’ (enzymes deactivation).

Mini-sequencing

Consist in a single base primer extension reaction in the presence of a DNA polymerase and a fluorophore-labeled terminator dideoxynucleotide (ddNTPs), the 3' of the primer was designed to be immediately upstream of the nucleotide of interest.

Ten primers (separated into two different SNaP primer mixes: MIX 3 and 4) were designed for mini-sequencing reaction (Table S2).

The reaction mix was prepared by adding 2.5 µL of MRRM (Applied Biosystem, Waltham, MA, USA), 0.5 µL SNaP primer mix and 2 µL of the purified PCR product, in a total volume of 5 µL. The solution was then placed in the thermal cycler and underwent to the following steps: (96° C for 10''; 50° C for 5''; 60° C for 30'') for 30 cycles; 10° C for ∞. At the end, the mini-sequencing reaction was again purified to remove unincorporated ddNTPs. FastAP 0.5 µL and 0.5 µL of distilled water were added to 1 µL of the minisequencing reaction; a two steps incubation: 37° C for 60" and 80° C for 15' was then performed. Two microliters of purified sample were then incubated at 95° C for five minutes with 0.5 µL of GeneScan 120 LIZ Size standard and 7.5 µL of formamide (Applied Biosystem, Waltham, MA, USA). The separation of fragments was carried out by capillary electrophoresis on an ABI PRISM 3130XL genetic analyzer with a 36 cm capillaries array, using the POP6 polymer (Applied Biosystems, Waltham, MA, USA). Results of the migrations were analyzed with GeneMapper v.4.1 software (Applied Biosystems, Waltham, MA, USA).

Cycle-sequencing

The mini-sequencing assay was validated by direct sequencing of PCR amplicons using modified Sanger method [1]; for this aim, 20 subjects of the control population were genotyped with both methods with a 100% concordance of the results.

The cycle sequencing mix was prepared as follow: 0.25 µL of the TRRM (Terminator Ready Reaction Mix), 0.875 µL of Big Dye Terminator 5x sequencing buffer, 1.625 µL of distilled water and 0.5 µL of the specific primer (forward or reverse) were added to the 2 µL of purified PCR sample; all reagents were supplied by Applied Biosystems, Waltham, MA, USA.

The solution was then placed in the thermal cycler to perform the following steps: 96° C. for 1'; (96° C for 10"; 50° C for 5"; 60° C for 4') for 30 cycles; 10° C for ∞. At the end of the sequencing cycles, the whole sample

underwent a further purification process with a QIAGEN chromatographic system (DyeEx 2.0 Spin Kit) following manufacturer instructions (Hilden, Germany). Purified samples were added to 15 μL of formamide and loaded in the ABI PRISM 3130xl Genetic Analyzer (Applied Biosystems, Waltham, MA, USA); generated fragments were resolved through an electrophoretic run in a 36 cm capillary array with the polacrylamide-based matrix POP6 (Applied Biosystems, Waltham, MA, USA). Running conditions were: injection time: 15 seconds, electrophoresis voltage: 1 kV, run lenght 50', running temperature: 50° C. Sequencing Analysis software supplied by the instrument manufacturer was used to evaluate the quality of the sequence produced. CLC Main Workbench 5 software (Qiagen, Hilden, Germany), was used to unveil the eventual presence of sequence variations. Wild type sequences, obtained from the Ensembl database (https://www.ensembl.org), were previously uploaded in the software CLC.

RNA extraction

About 10 milligrams of normal, non-tumoral tissue was obtained from each patient from the margins of resection. Tissue samples were immediately immerged in 1mL of Trizol reagent and stored at -80° C until the moment they have been processed. Tissue was homogenized before extraction; RNA was obtained using Trizol Reagent (Invitrogen, Waltham, MA, USA) protocol, following manufacturer instructions. RNA from each available tissue was obtained in a volume of 20 μL. Extracted RNA was then quantified using Qubit fluorimetric assay (Invitrogen, Waltham, MA, USA).

Reverse Transcription

A DNase treatment was performed to remove any contaminating genomic DNA: 4 μg of total RNA were incubated with 2.2 units (0.7 μL) of DNase I (New England Biolabs, Ipswich, MA, USA) and 2 μL of 10x DNase1 reaction buffer at 37° C for 10' in a final volume of 20 μL. Next, 2.2 μL of 1x EDTA (5mM, pH = 8) (Sigma-Aldrich, St Louis, MO, USA) were added to the samples; an incubation at 75° C for 10' was then performed to inactivate the enzyme. Digested sample (5.5 μL, corresponding to 1mg of RNA) were added to 1 μL of iScript Reverse Transcriptase, 4 μL of 5x iScript Reaction mix and 9.5 μL of distilled water (all reagents from iScript cDNA Synthesis Kit – Bio-Rad, Hercules, CA, USA) in a final volume of 20 μL. The reaction was placed in a PTC100 thermal cycler (Bio-Rad, Hercules, CA, USA) and followed the protocol:

25° C for 5’; 42° C for 30’; 85° C for 5’. As quality control, an amplification of GAPDH housekeeping gene was performed with 2 µL of cDNA, 5.7 µL of distilled water, 2.1 µL of dNTPs 1.25 mM (Thermo Fisher Scientific, Waltham, MA, USA), 3 µL of Buffer Mix Reaction 5x (Promega, Madison, WI, USA), 0.9 µL $MgCl_2$ 25 mM (Promega, Madison, WI, USA), 0.1 µL di Gotaq DNA polymerase 5 U/mL (Promega, Madison, WI, USA) and 0.6 µL of the GAPDH forward primer (5’-CCCTTCATTGACCTCAACTACATG-3’) and 0.6 µL of the reverse primer (5’-TGGGATTTCCATTGATGACAAGC-3’) in a total volume of 15 µL. Thermal cycling were set as follow: 95° C for 2’, (94° C for 45’’; 60° C for 1.5’; 70° C for 2.5’) for 28 cycles; 72° C for 7’; 10° C ∞. Amplicons, 3.3 µL of each sample, were run in a 2% agarose gel electrophoresis (Bio-Rad, Hercules, CA, USA) and distinguished by their size using a 50 bp molecular weight standard (Thermo Fisher, Waltham, MA, USA).

Digital droplet PCR (ddPCR)

For quantitative ADH7 and ALDH2 expression analysis, Droplet Digital PCR (ddPCR) was performed starting from cDNA of selected samples. TaqMan gene expression assay (ADH7 code 4448892, ID Hs00609447_m1; ALDH2 code 4448892, ID Hs01007998_m1; Thermo Fisher, Waltham, MA, USA) with the TaqMan probe FAM dye-labeled were used, according to the manufacturer’s instructions. Final reaction volume was 20 µL: 1 µL of cDNA mix, 11 µL of 2x ddPCR Supermix for probes (no dUTP), 1 µL of specific TaqMan probe assay, 9 µL of H_2O. According to manufacturer instructions, 70 µL of droplet generation oil for probes was added. The water-in-oil droplet emulsion was prepared by the QX200 Droplet Generator. The amplification step was performed using 40 µL of emulsion in a C1000 thermal cycler using the following protocol: 95° C for 10’; (94° C for 30″; 60° C for 1′) for 45 cycles, followed by 98° C for 10’. The ddPCR reactions were analyzed by the QX200 Droplet Reader and QuantaSoft software version 1.7.4 (all instruments, reagents and software were from Bio-Rad, Hercules, CA, USA).

Table S4. Allelic frequencies of investigated polymorphisms in laryngeal cancer patients and controls.
CI, confidence interval and OR, odds ratio. In bold are indicated P values <0.05 (significant threshold after Bonferroni correction).

Polymorphisms	Allele	Ctrls (N=20) N (frequency)	Tumor (N=21) N (frequency)	P	OR	95% CI
ADH1B – rs1229984	C	37 (0.925)	41 (0.976)	0.3535	3.324	0.3312 to 33.37
	T	3 (0.075)	1 (0.024)			
ADH1B – rs2066702	G	37 (0.925)	42 (1.00)	0.1116	7.933	0.3967 to 158.6
	A	3 (0.075)	0 (0.00)			
ADH1C – rs1693482	C	26 (0.65)	30 (0.714)	0.6366	1.346	0.5296 to 3.422

	T	14 (0.35)	12 (0.286)			
ADH7 – rs894363	C	24 (0.60)	21 (0.50)	0.3842	0.6667	0.2778 to 1.600
	T	16 (0.40)	21 (0.50)			
ADH7 – rs1154470	G	24 (0.60)	24 (0.571)	0.8258	0.8889	0.3689 to 2.142
	A	16 (0.40)	18 (0.429)			
ADH7 – rs1154468	T	24 (0.60)	24 (0.571)	0.8258	0.8889	0.3689 to 2.142
	A	16 (0.40)	18 (0.429)			
ADH7 – rs1573496	C	37 (0.925)	40 (0.952)	0.6718	1.622	0.2565 to 10.25
	G	3 (0.075)	2 (0.048)			
ADH7 – rs971074	C	34 (0.85)	40 (0.952)	0.1504	3.529	0.6682 to 18.64
	T	6 (0.15)	2 (0.048)			
ADH7 – rs1154461	C	25 (0.625)	25 (0.595)	0.8238	0.8824	0.3629 to 2.145
	G	15 (0.375)	17 (0.405)			
ALDH2 – rs671	G	40 (1.00)	41 (0.977)	>0.9999	0.3416	0.01351 to 8.632
	A	0 (0.00)	1 (0.023)			

Table S5. Genotypic frequencies of investigated polymorphisms in the enrolled patients and controls.
CI, confidence interval and OR, odds ratio. In bold are indicated P values <0.05 (significant threshold after Bonferroni correction).
*Individuals with at least one variant allele were grouped in one category and compared against the homozygous wild type genotype

Polymorphisms	Genotype*	Ctrls (N=20) N (frequency)	Tumor (N=21) N (frequency)	P	OR	95% CI
ADH1B – rs1229984	CC	17 (0.85)	20 (0.952)	0.3433	3.529	0.3353 to 37.15
	TT+CT	3 (0.15)	1 (0.048)			
ADH1B – rs2066702	GG	17 (0.85)	21 (1.00)	0.1069	8.600	0.4157 to 177.9
	AA+GA	3 (0.15)	0 (0.00)			
ADH1C – rs1693482	CC	9 (0.45)	10 (0.476)	>0.9999	1.111	0.3252 to 3.796
	TT+CT	11 (0.55)	11 (0.524)			
ADH7 – rs894363	CC	8 (0.40)	4 (0.190)	0.1809	0.3529	0.08621 to 1.445
	TT+CT	12 (0.60)	17 (0.810)			
ADH7 – rs1154470	GG	8 (0.40)	6 (0.286)	0.5204	0.6000	0.1631 to 2.207
	AA+GA	12 (0.60)	15 (0.714)			
ADH7 – rs1154468	TT	8 (0.40)	6 (0.286)	0.5204	0.6000	0.1631 to 2.207
	AA+TA	12 (0.60)	15 (0.714)			
ADH7 – rs1573496	CC	17 (0.85)	19 (0.905)	0.6628	1.676	0.2495 to 11.27
	GG+CG	3 (0.15)	2 (0.095)			
ADH7 – rs971074	CC	14 (0.70)	19 (0.905)	0.1300	4.071	0.7126 to 23.26
	TT+CT	6 (0.30)	2 (0.095)			
ADH7 – rs1154461	CC	9 (0.45)	6 (0.286)	0.3408	0.4889	0.1341 to 1.782
	GG+CG	11 (0.55)	15 (0.714)			
ALDH2 – rs671	GG	20 (1.00)	20 (0.952)	>0.9999	0.3333	0.01281 to 8.671
	AA+GA	0 (0.00)	1 (0.048)			

Reference:

1 Sanger F, Nicklen S, Coulson AR. DNA sequencing with chain-terminating inhibitors. Proc Natl Acad Sci U S A 1977;74:5463–7. doi:10.1073/pnas.74.12.5463.
